# Tuberculosis of the elbow mimicking rheumatoid arthritis

**DOI:** 10.14744/nci.2020.68916

**Published:** 2021-12-13

**Authors:** Sadettin Uslu

**Affiliations:** Department of Rheumatology, Omer Halisdemir University Bor Physical Medicine and Rehabilitation, Training and Research Hospital, Nigde, Turkey

**A** 63-year-old woman presented with a 6-month history of a painful swollen left elbow joint. Medical inquiry was negative for preceding infections or trauma and there were no constitutional symptoms. Physical examination of the elbow revealed tenderness and swelling. The white cell count was normal, while the erythrocyte sedimentation rate (48; range, 0–20 mm/h) and C-reactive protein levels (22; range, 0–5 mg/l) were increased. Rest of the tests including RF, anti-CCP, and ANA were negative and uric acid level was normal. X-ray of the elbow showed juxta-articular osteoporosis, erosion, and narrowing of the joint space (Phemister’s triad; Fig. 1). Synovial aspiration showed increased leukocytes (9300/mm3) without crystals. Histopathological examination of synovial biopsy showed granulomatous inflammation and tissue culture revealed Mycobacterium tuberculosis (TB). Ziehl-Neelsen stain showed acid-fast bacilli, confirming the diagnosis of TB arthritis. There was no history of other chronic disease or contact with individuals having TB. Findings on chest radiograph and sputum culture were normal. The diagnosis of TB monoarthritis was established and the patient was started on four-drug anti-TB regimen. In our case, 9 months of TB treatment resulted symptomatic control of pain and swelling.

**Figure 1.**
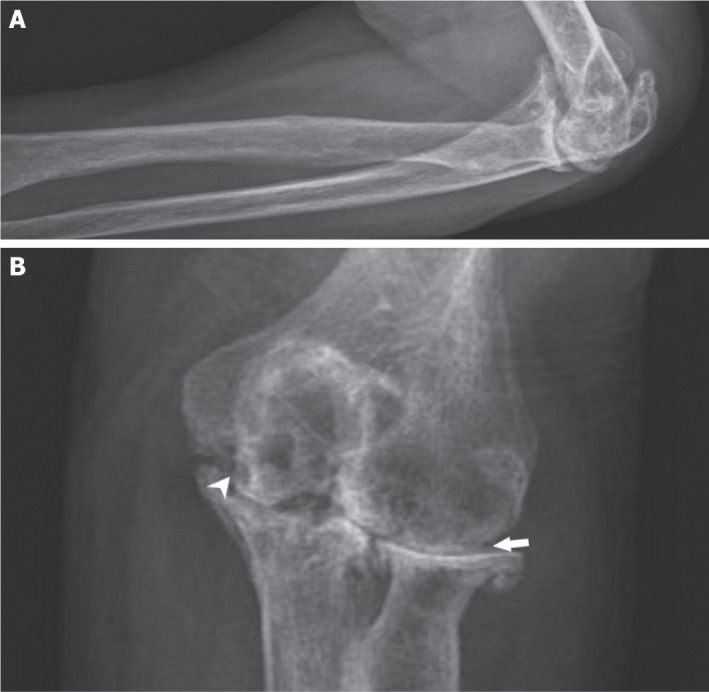
**(A)** Lateral and **(B)** AP X-ray of the left elbow, showing juxta-articular osteoporosis, erosion (arrowhead) and narrowing of the joint space (arrow).

Skeletal TB accounts for 5–15% of extra-pulmonary TB and is often restricted to the spine and weight-bearing joints such as the hip and knee. TB of the elbow comprises less than 5% of all skeletal TB cases.^[1]^ Radiographic findings of the affected joint are non-specific, however; Phemister’s triad including periarticular osteoporosis, erosions, and narrowing of the cartilage space could be seen.^[2]^ The differential diagnoses of skeletal TB include pyogenic arthritis, rheumatoid arthritis, sarcoid arthritis, gout, pigmented villonodular synovitis, and tumors. Our case is a good example to consider osteoarticular TB in the differential diagnosis of chronic monoarticular joint pain and swelling even if significant risk factors are absent.

## References

[1] Aggarwal A, Dhammi I (2006). Clinical and radiological presentation of tuberculosis of the elbow.. Acta Orthop Belg.

[2] Uslu S, Balcı A, Sarı İ (2020). Tuberculosis monoarthritis of the wrist mimicking rheumatoid arthritis.. Eur J Rheumatol.

